# Analysis of Beta-Dystroglycan in Different Cell Models of Senescence

**DOI:** 10.3390/ijms26167726

**Published:** 2025-08-10

**Authors:** Guadalupe Elizabeth Jimenez-Gutierrez, Tania Ivette Zavaleta-Vásquez, Jessica Alexandra Lizcano-Meneses, Ian Alain Garcia-Aguirre, Marco Samuel Laredo-Cisneros, Jonathan J. Magaña, Steve J Winder, Joaquín Cordero-Martínez, Bulmaro Cisneros

**Affiliations:** 1 Laboratorio de Medicina Genómica, Instituto Nacional de Rehabilitación Luis Guillermo Ibarra Ibarra, Mexico City 14389, Mexico; gejimenezg@inr.gob.mx (G.E.J.-G.); magana.jj@tec.mx (J.J.M.); 2 Departamento de Genética y Biología Molecular, Centro de Investigación y de Estudios Avanzados del Instituto Politécnico Nacional, Mexico City 07360, Mexico; tania.zavaleta@cinvestav.mx (T.I.Z.-V.); jessicaalexandralm@ufps.edu.co (J.A.L.-M.); marco32x@hotmail.com (M.S.L.-C.); 3 Departamento de Bioingeniería, Escuela de Ingeniería y Ciencias, Tecnologico de Monterrey, Campus Ciudad de México, Mexico City 14380, Mexico; ian.garcia@tec.mx; 4 School of Biosciences, The University of Sheffield, Western Bank, Sheffield S10 2TN, UK; s.winder@shef.ac.uk; 5 Laboratorio de Bioquímica Farmacológica, Departamento de Bioquímica, Escuela Nacional de Ciencias Biológicas, Instituto Politécnico Nacional, Mexico City 11340, Mexico

**Keywords:** β-DG, senescence, intracellular domain (ICD), nuclear lamina

## Abstract

The functional diversity of β-dystroglycan is attributable to its dual distribution, the plasma membrane, and the nucleus. In the plasma membrane, β-DG is a component of the dystrophin-associated protein complex. In the nucleus, β-DG assembles with the nuclear lamina and emerin. Recent findings indicate a role for β-DG in senescence, as its knockout in C2C12 myoblasts induces genomic instability and promotes the senescent state. This study analyzed the behavior of β-DG in three distinct models of senescence: chronologically aged fibroblasts, sodium butyrate (NaBu)-induced senescent fibroblasts, and fibroblasts from a Hutchinson–Gilford progeria syndrome (HGPS) patient. β-DG was found mainly in the nucleus in all the senescent cell types, with a certain mislocalization to the cytoplasm in HGPS and NaBu-treated fibroblasts. Furthermore, the full-length β-DG (43 kDa) and the cleaved intracellular domain (ICD; ~26 kDa) were identified. The ICD level increased in aged fibroblasts, but its yield was poor or virtually nonexistent in NaBU-induced and HGPS fibroblasts, respectively. Remarkably, β-DG was sequestered by progerin in HGPS cells, hindering its interaction with lamin A. In summary, the observed alterations in β-DG may be associated with the senescent state, and such findings will serve for future studies aimed at elucidating its role in senescence.

## 1. Introduction

The process of human aging is defined as a decline in the individual’s physiological capacity, encompassing both physical and cognitive functions [1]. Additionally, it represents a significant risk factor for a range of chronic degenerative diseases, including cardiovascular, musculoskeletal, metabolic, and neurodegenerative diseases [1,2]. At the cellular level, deterioration resulting from multiple cell divisions (replicative aging), the accumulation of DNA damage, or various types of stress caused by the expression of oncogenes or the action of chemical agents that affect chromatin organization, ultimately leads to irreversible cell cycle arrest in the G0/G1 phase, a phenomenon known as cellular senescence [3]. A number of cellular alterations have been identified as hallmarks of aging, including telomere shortening, mitochondrial dysfunction, alterations in proteostasis, and deterioration of nuclei and nucleoli morphology and function [4]. Despite the extensive research conducted over the past decade, further investigation is necessary to elucidate in depth the mechanisms underlying senescence and to identify novel proteins that may play a role in preventing aging.

In this context, β-dystroglycan (β-DG) emerges as a promising candidate due to its involvement in various senescence-related functions. β-DG is an essential transmembrane protein that serves as a bridge between the extracellular matrix and the actin cytoskeleton, thereby providing structural stability to the plasma membrane and regulating signal transduction from the extracellular space to the cytoplasm of the cell [5]. This is facilitated by its interaction with alpha-DG in the extracellular matrix and dystrophin in the cytoplasm [5]. On the other hand, β-DG plays a pivotal role in maintaining the structural integrity of the nuclear envelope (NE) through its interactions with lamins A/C, B1, and emerin [6]. It is noteworthy that β-DG undergoes proteolytic cleavage by matrix metalloproteinase (MMP)-2 and -9, resulting in the liberation of its extracellular domain; it is postulated that the remaining fragment, comprising the transmembrane stub and the cytoplasmic portion, is subsequently processed by γ-secretase, leading to the delivery of an intracellular domain (ICD) [7]. The ICD exerts a negative regulatory effect on ribosomal DNA transcription through its interactions with B23/nucleophosmin and upstream binding factor (UBF), thereby modulating the cellular response to nucleolar stress [8].

The significance of β-DG in the study of senescence was previously evidenced. The knockout of DG expression in C2C12 mouse myoblasts was found to result in alterations in nuclear morphology and a reduction in the levels of nuclear envelope proteins lamin A and emerin [9]. Furthermore, the absence of DG resulted in defective mitosis and genomic instability, which ultimately led to the emergence of senescence hallmarks [9]. Although these findings indicate that β-DG plays a crucial role in protecting cells against stress-induced senescence, further investigation of these findings in cellular models of senescence is warranted. In this study, we analyzed the behavior of β-DG in three distinct cellular models of senescence. These comprised a primary fibroblast culture derived from a normal individual, which was induced to senescence through treatment with sodium butyrate [10]; a primary fibroblast culture derived from a healthy aged individual (96 years of age); a well stablished senescent model that exhibits most of the ubiquitous hallmarks of aging [11]; and a primary fibroblast culture derived from a patient with Hutchinson–Gilford progeria syndrome (HGPS) [12].

## 2. Results

### 2.1. Analysis of β-DG in Physiological Aged Primary Fibroblasts

**Figure 1:**
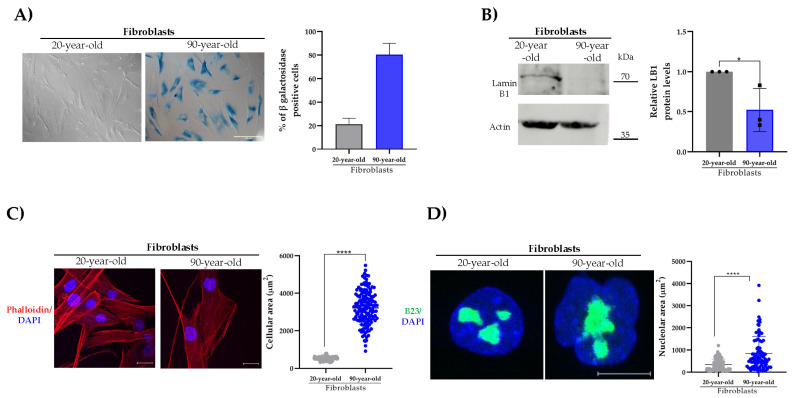
**Analysis of senescence hallmarks in young and aged primary fibroblasts**. (**A**) Senescent cells were identified in young and aged fibroblasts by SA-(β-gal) activity staining. Representative images were captured using bright-field microscopy. The percentage of senescent-positive cells was quantified from three separate experiments (*n* = 100 cells for each condition) using NIS elements software (Nikon, Tokyo, Japan). Scale bar = 150 μm. (**B**) Lysates from young and aged fibroblasts were analyzed by SDS-PAGE/WB using primary antibodies against lamin B1 and β-actin (loading control). Representative images from three independent experiments are shown. Statistical differences were assessed using Student’s *t*-test (* *p* < 0.05). Data represent the mean ± SEM. (**C**) Cells grown on coverslips were labeled with DAPI and phalloidin to visualize nuclei and the actin-based cytoskeleton, respectively. Representative images from three independent assays are shown. Scale bar = 50 μm. Right panel: the cellular area was estimated using ImageJ software (v1.54k), with significant differences determined by a non-parametric Mann–Whitney U test. Data are the mean ± SEM (* *p* < 0.05). (**D**) Cells cultured on coverslips were immunolabeled for B23 to visualize nucleoli and counterstained with DAPI to label nuclei. Scale bar = 10 μm. Right panel: the nucleolar area was determined using ImageJ software (v1.54k) and the non-parametric Mann–Whitney U test. Data corresponds to the mean ± SEM (* *p* < 0.05; **** *p* ≤ 0.0001).

To analyze the behavior of β-DG in a cellular model of natural aging, primary cultures of fibroblasts from donors aged 20 and 90 years were used. Firstly, the senescent phenotype of the cell cultures was characterized. We found that the activity of the Senescence-Associated β-galactosidase enzyme SA-(β-gal), a widely used criterion for identifying senescent cells, was significantly increased in aged fibroblasts, compared to young fibroblasts (~80% versus ~20%) (Figure 1A). Owing to the role of lamin B1 in regulating heterochromatin organization and nuclear architecture, the downregulation of this protein is another molecular indicator of cellular senescence. A decline in lamina B1 levels was observed in aged fibroblasts by immunoblotting assays (Figure 1B). Two additional hallmarks of senescent cells were identified in most of the aged fibroblasts: senescent cell morphology (expanded flattened cell morphology) and nucleolar expansion (1–2 prominent nucleoli per cell), as revealed by phalloidin staining of F-actin and immunolabeling of the nucleolar protein B23, respectively (Figure 1C,D). Collectively, these results show that the analyzed senescent hallmarks predominate in aged fibroblast cultures, validating our cell model of natural aging.

**Figure 2:**
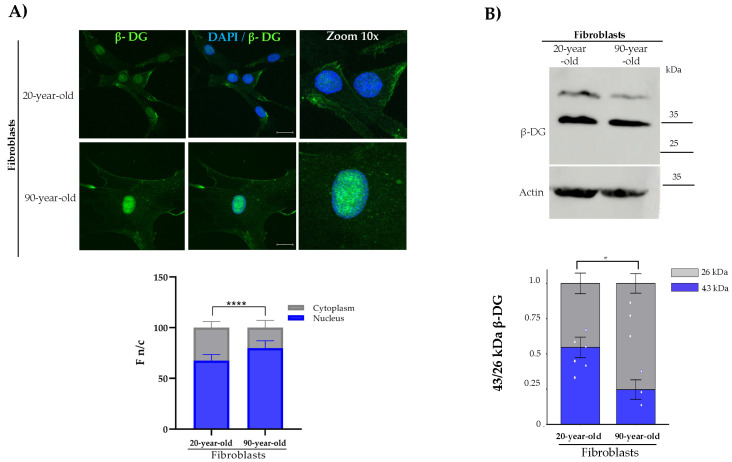
**Analysis of β-DG localization and expression in young and aged primary fibroblasts.** (**A**) Cells grown on coverslips were immunolabeled with primary antibodies against β-DG and counterstained with DAPI to visualize nuclei. Typical images from three separate experiments are shown. The F n/c ratio of β-DG was calculated from three independent experiments using ImageJ software (*n* = 100 cells per experimental condition), and significant differences were determined by Mann–Whitney U test (* *p*< 0.001) (scale bar = 20 μm). (**B**) β-DG protein expression was assessed by SDS-PAGE/WB using specific antibodies to β-DG and β-actin (loading control). Representative blots from three independent experiments are shown. Right panel: the relative levels of β-DG were quantified using Student’s *t*-test (* *p* < 0.05; **** *p* ≤ 0.0001).

Subcellular localization and protein expression of β-DG were then evaluated in these cell cultures. A predominant nuclear localization of β-DG, with comparatively lower intensity in the cytoplasm, was observed in both young and aged fibroblasts; however, the nuclear staining of the protein was found to be even higher in aged cells (Figure 2A). On the other hand, the immunoblotting assays revealed the presence of two β-DG fragments (43 kDa and 26 kDa) in both young and aged fibroblasts (Figure 2B), which probably correspond to the full-length protein and the ICD cleavage fragment. It is worth noting that the proteolytic cleavage undergone by β-DG to generate ICD occurred at a higher frequency in aged fibroblasts.

### 2.2. Evaluation of β-DG in Senescence-Induced Fibroblasts

**Figure 3:**
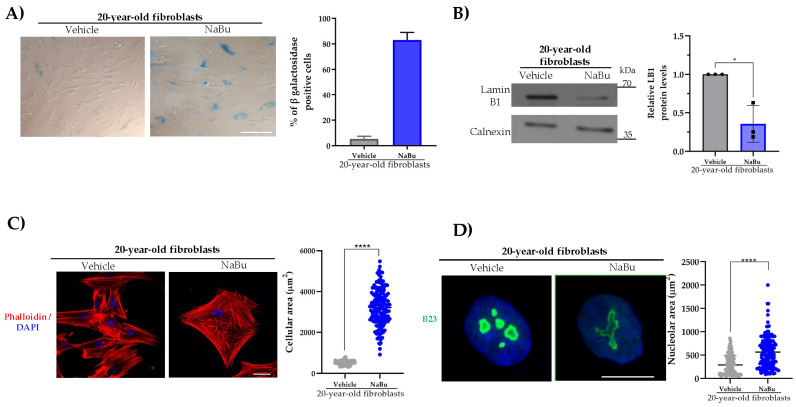
**Hallmarks of aging in Na-Bu-induced senescent fibroblasts**. (**A**) Senescent cells were identified in control and senescence-induced fibroblasts by quantifying SA β-gal activity. Representative images were captured using bright field microscopy. The percentage of senescent-positive cells was scored from three independent experiments (*n* = 50 cells for each condition) using NIS elements software (Nikon). Scale bar = 150 μm. (**B**) Lysates from the control and senescence-induced fibroblasts were analyzed by SDS-PAGE/WB using primary antibodies against lamin B1 and calnexin (loading control). Representative images from three independent experiments are shown. Statistical differences were assessed using Student’s *t*-test (* *p* < 0.05 in comparison to the control fibroblasts). Data presented as mean ± SEM. (**C**) Cells were labeled with DAPI and phalloidin to visualize their nuclei and actin-based cytoskeleton, respectively. Representative images are shown. Scale bar = 20 μm. Right: the cellular area was estimated using ImageJ software with significant differences determined by a non-parametric Mann–Whitney U test. Data are presented as mean ± SEM; * *p* < 0.05 compared to the control fibroblasts. (**D**) Nucleolar morphology was analyzed by immunolabeling for B23 and labeled with DAPI staining. Scale bar = 10 μm. Right: nucleolar area was determined by the non-parametric Mann–Whitney U test. Data are presented as mean ± SEM; * *p* < 0.05 and **** *p* ≤ 0.0001 compared to the control fibroblasts.

To determine whether the induction of primary fibroblasts into senescence exerts an effect on the subcellular distribution and protein levels of β-DG, fibroblasts from a young donor (20 years of age) were treated with sodium butyrate (NaBu) for a period of five days. NaBu is a histone deacetylase inhibitor that has been shown to irreversibly halt cell cycle progression, thereby promoting senescence [13]. To confirm effective induction of senescence, different senescent hallmarks were evaluated. The number of SA-(β-gal)-positive cells was found to significantly increase (~80%) in NaBu-treated fibroblasts, compared to vehicle-treated fibroblasts (~5%) (Figure 3A). In accordance with the acquisition of a senescent phenotype upon NaBu treatment, a marked decrease in the content of lamin B1 protein was observed (Figure 3B), and concurrently, the cellular and nucleolar areas exhibited a significant augmentation in these senescent-induced fibroblasts (Figure 3C,D). Furthermore, the protein level of the cyclin-dependent kinase inhibitor p21^WAF1/CIP1^ was evaluated, given its ability to inhibit cyclin E/CDK2 and cyclin A/CDK2. This, in turn, results in the hypophosphorylation of pRB, thereby preventing the release of E2F transcription factors and blocking the cell cycle’s entry into the S phase [10]. As anticipated, p21 levels were observed to increase in NaBu-induced senescent fibroblasts (Supplementary Figure S1A).

**Figure 4:**
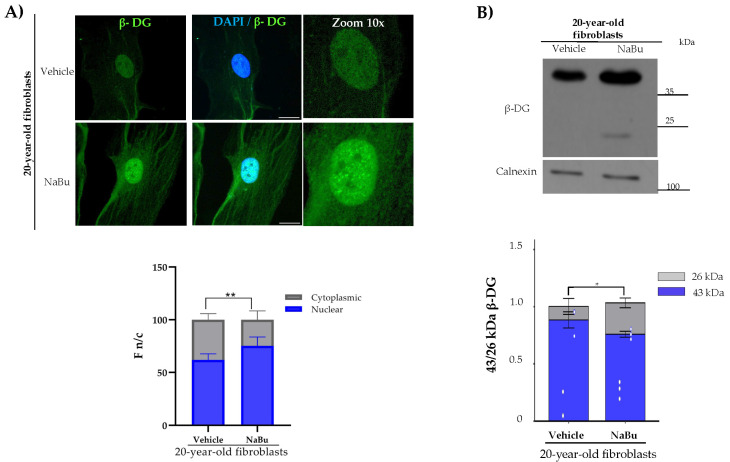
**β-DG distribution and protein expression in Na-Bu-induced senescent fibroblasts.** (**A**) To analyze β-DG expression, cells were immunolabeled with the corresponding primary antibody and counterstained with DAPI. The F n/c ratio of β-DG was scored from three separate experiments using ImageJ software (*n* = 300 cells per experimental condition), and significant differences were determined by Mann–Whitney U test (* *p* < 0.001). Scale bar = 10 μm. (**B**) β-DG protein expression was assessed by SDS-PAGE/WB using specific antibodies against β-DG and calnexin (loading control). Representative blots are shown. Right: relative protein expression was quantified using Student’s *t*-test (* *p* < 0.05; ** *p* ≤ 0.01 in comparison to the control fibroblasts).

With regard to the analysis of β-DG, we observed prominent nuclear localization in both fibroblast cultures; however, the immunofluorescence intensity of β-DG throughout the cell was higher in NA-Bu-induced senescent fibroblasts. Western blot analysis revealed that protein lysates from the control fibroblasts contained only the full-length β-DG fragment, while old fibroblast protein lysates contained the full fragment and a small portion of the ICD fragment (Figure 4A).

### 2.3. Analysis of β-DG in HGPS Fibroblasts

**Figure 5:**
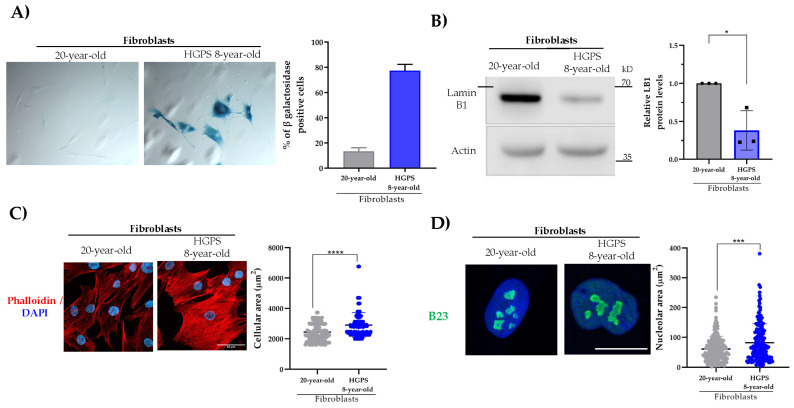
**Characterization of the senescent phenotype of HGPS fibroblasts**. (**A**) Control (20-year-old fibroblasts) and HGPS fibroblasts were subjected to staining for β-gal enzyme activity. The percentage of senescent-positive cells was calculated and scored from three independent experiments (*n* = 100 cells for each condition) using NIS elements software (Nikon). Scale bar = 150 μm. (**B**) The level of lamin B1 (LB1) was assessed by Western blotting in the control and HGPS lysates in three independent experiments. (**C**) The cellular morphology of the control and HGPS fibroblasts was evaluated by labeling the actin-based cytoskeleton with phalloidin. Nuclei were visualized by DAPI staining. Scale bar = 50 μm. (**D**) The control and HGPs fibroblasts were subjected to immunostaining with B23 to decorate nucleoli, and the nuclei were counterstained with DAPI. Scale bar = 10 μm. (**C**,**D**) The cellular and nucleolar areas were determined by the non-parametric Mann–Whitney U test. Data are presented as mean ± SEM; * *p* < 0.05; *** *p* ≤ 0.001; **** *p* ≤ 0.0001 compared to the control fibroblasts.

Cultured dermal fibroblasts derived from a patient with HGPS were utilized to analyze β-DG in a cell model of pathological aging [14]. The mutant variant of lamin A, termed progerin, leads to nuclear envelope (NE) destabilization and DNA damage, which collectively contribute to the senescent phenotype observed in these cells [15]. As anticipated, a significant increase in SA-β-gal-positive cells (Figure 5A), accompanied by a decrease in lamin B1 protein levels (Figure 5B) and increased p21^WAF1/CIP1^ protein levels (Supplementary Figure S1B), was found in HGPS fibroblasts, compared with the control fibroblasts (20-year-old fibroblasts). Furthermore, HGPS fibroblasts exhibited an enlarged flattened cell morphology that contrasted with the fusiform morphology of the control fibroblasts (Figure 5C). Finally, expanded nucleoli, a hallmark of senescent cells, were observed exclusively in the culture of HGPS fibroblasts upon immunostaining with the nucleolar protein marker B23 (Figure 5D). Confocal microscopy images revealed a predominant nuclear localization of β-DG in both the control and HGPS fibroblasts.

**Figure 6:**
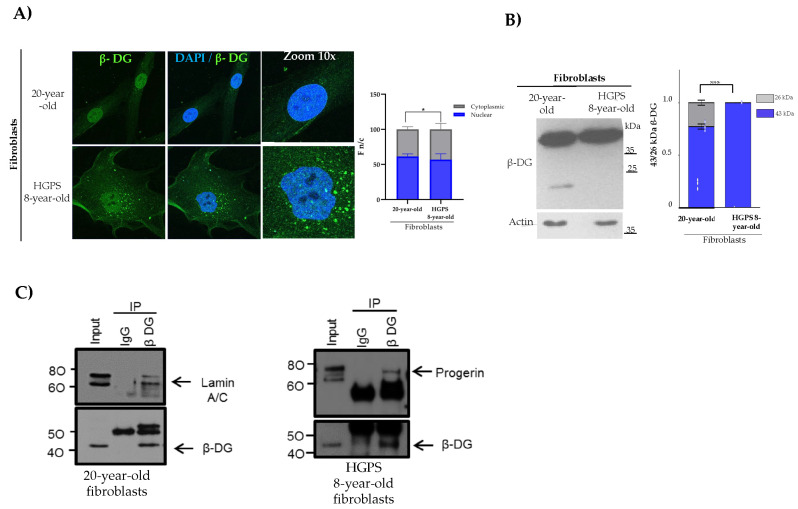
**β-DG subcellular localization and protein expression in HGPS fibroblasts.** (**A**) The control and HGPS fibroblasts were immunostained using β-DG antibodies. Nuclei were visualized using DAPI. The F n/c ratio of β-DG was calculated from 50 cells per experimental condition and from three independent experiments using ImageJ software. Significant differences were determined by Mann–Whitney U test (* *p* < 0.05). Scale bar = 10 μm. (**B**) The protein level of β-DG was analyzed by WB using specific antibodies directed to β-DG and β-actin (loading control). A typical blot of three separate experiments is shown. Right: relative protein expression was quantified using Student’s *t*-test (*** *p* ≤ 0.001) in comparison to the control fibroblasts. (**C**) Immunoprecipitation (IP) assays were carried out in the control and HGPS protein lysates using anti-β-DG antibodies, and the eluted fraction was further analyzed with specific antibodies against lamin A/C or progerin. None of the specific bands were observed in the immunoprecipitates obtained with irrelevant IgG (IgG0). Representative immunoblots from two independent experiments are shown.

However, the cytoplasmic signal of β-DG was clearly more intense in the latter cells (Figure 6A). On the other hand, the analysis of protein lysates using WB assays revealed the presence of the full-length β-DG fragment (43 kDA) and a faint band consistent with the ICD fragment (26 kDA) in the control fibroblasts. In contrast, HGPS protein lysates contained solely the β-DG full-length protein (Figure 6B). Given the ability of β-DG to bind to lamin A in C2C12 myoblasts, we hypothesized that this protein may also interact with progerin. Immunoprecipitation assays utilizing β-DG antibodies demonstrated the ability of β-DG to bind to lamin A/C in the control fibroblasts; nonetheless, β-DG displayed a selective binding affinity for progerin in HGPS fibroblasts (Figure 6C).

## 3. Discussion

In this study, the subcellular distribution and protein level of β-DG were evaluated in three models of cellular senescence in order to ascertain how β-DG responds to different stimuli that cause senescence. Cellular senescence is defined as the arrest of the cell cycle due to the accumulation of genomic damage or the exposure of the cell to various types of stress, including oxidative stress, oncogenic signaling, and UV exposure [16]. However, the specific events that lead to cellular senescence are determined by the initial stimulus.

The dermal fibroblast cell culture derived from an aged individual should predominantly comprise senescent cells, thereby differentiating it from that of a younger subject. Because the skin manifests evident signs of aging due to chronological accumulation of biophysical changes [17], thus, fibroblasts undergo replicative aging and may reach senescence [17]. On the other hand, sodium butyrate (NaBu) has been shown to induce cell senescence through epigenetic regulation, as it exerts an inhibitory effect on histone deacetylases (HDAC). It has been established that the accumulation of acetylated histones triggers senescence, at least in part, by upregulating and stabilizing the cell cycle regulatory proteins p21^WAF1/CIP1^ and p53 [13]. Finally, the progression of HGPS fibroblasts into cellular senescence is attributable to the toxic action of progerin, a mutant variant of lamin A. The progerin attaches erroneously to the nuclear envelope, thereby altering the structure of the nuclear lamina, which in turn exerts downstream deleterious effects on a variety of cellular processes, including gene regulation, mitochondrial function, telomere homeostasis, and the DNA damage response [15]. Ultimately, these effects culminate in cell cycle arrest. All three senescent cell cultures analyzed exhibited hallmarks of senescence, namely SA-β-gal-positive cells, enlarged and flattened cellular morphology (senescent morphology), nucleolar expansion, and lamin B1 protein depletion.

The analysis of β-DG showed that the protein displayed a predominantly nuclear distribution in the three senescent cell models analyzed. However, a higher signal for the protein in the cytoplasm was observed in NaBu-induced senescent fibroblasts and HGPS fibroblasts compared to the control fibroblasts. Owing to the fact that β-DG harbors both nuclear import (NLS) and nuclear export (NES) motifs, which act in concert with importins α1/βb2 and exportin CRM1, respectively, to regulate its nucleocytoplasmic trafficking, it is conceivable that these mechanisms may be altered in the senescent cells. Specifically, it has been demonstrated that HGPS fibroblasts overexpress CRM1, which results in an exacerbated export to the cytoplasm of NES-containing nuclear proteins (i.e., β-DG), thereby altering their proteostasis and ultimately leading them to senescence [18]. The interaction of β-DG with the NE proteins lamina B1, lamina A/C, and emerin contributes to the stability of the nuclear envelope. Therefore, it can be argued that the presence of β-DG in the nucleus could mitigate the damage caused by partial loss of lamina B1 in senescent cells. Additional experiments are necessary to evaluate this hypothesis. It is worth noting that β-DG was found to bind to lamin A/C in normal fibroblasts but exclusively to progerin in HGPS fibroblasts. As progerin exerts its deleterious effects by interfering with the function of lamin A [19], the observed loss of interaction between β-DG and lamin A may be a contributing factor to the NE-associated alterations exhibited by these cells.

We identified two β-DG proteins in the cell cultures analyzed, the 43 kDa full-length protein and the 26 kDa ICD fragment. The cleavage of ICD is induced by various nucleolar stressors, including oxidative stress, acidosis, and UV irradiation [7,8]. Following its release, ICD is targeted to the nucleolus, where it functions as a negative regulator of ribosomal DNA (rDNA) transcription, thereby safeguarding cells against nucleolar stress [8]. The levels of the ICD fragment increased solely in the culture of old fibroblasts. In contrast, the generation of this cleaved fragment was scarce or virtually absent in NaBu-induced senescent fibroblasts and HGPS fibroblasts. Owing to the fact that the nucleolar expansion that occurs in senescent cells is due to an increased ribosome biogenesis and activity [20], it appears that the absence or low quantity of the ICD fragment prevents the senescent cells (HGPS and Na-Bu-treated fibroblasts) from counteracting such exacerbated rDNA synthesis. Further experiments are necessary to assess this hypothesis.

## 4. Materials and Methods

### 4.1. Cell Culture and Treatments

The primary human dermal fibroblasts derived from two healthy donors aged 20 (GM03440) and 96 years (AG04059), as well as from an 8-year-old HGPS patient, who is a carrier of the classic G608G splice site mutation at the LMNA gene (AG11513), were acquired from the Coriell Institute for Medical Research (Camden, NJ, USA). The fibroblasts were cultured at 37 °C in a humidified 5% CO_2_ atmosphere in Eagle’s Minimal Essential Medium (MEM; Invitrogen, Carlsbad, CA, USA) supplemented with 15% (v/v) fetal bovine serum (Invitrogen, Thermo Fisher Scientific, Waltham, MA, USA), 100 U/mL penicillin, 10 µg/mL streptomycin, and 1 mM sodium pyruvate (Sigma, Saint Louis, MO, USA). All experiments were conducted prior to the 16th cell culture passage. Where indicated, fibroblast cultures were treated for five days with sodium butyrate (Cat. No. B5887, Sigma-Aldrich, St. Louis, Missouri, United States) diluted in PBS 1X to a final concentration of 10 mM.

### 4.2. Antibodies

The following primary antibodies were used. The mouse monoclonal antibodies included β-dystroglycan (MANDAG2), lamin B1 (Ab16048; Abcam, Cambridge, UK), actin (sc-37642; Santa Cruz Biotechnology, Dallas, TX, USA), and B23/NPM1 (Cat. No. 32-5200; Invitrogen, Carlsbad, CA, USA). Rabbit polyclonal antibody against calnexin (sc-11397; Santa Cruz Biotechnology, Dallas, TX, USA) was also used.

### 4.3. Immunofluorescence and Confocal Microscopy Analysis

Fibroblasts were cultured on coverslips and fixed with 4% paraformaldehyde for 10 min, permeabilized with 0.2% Triton X-100, and blocked with 2% bovine serum albumin (BSA) for 20 min. The coverslips were incubated overnight at 4 °C with the respective primary antibodies. The next day, the coverslips were washed two times with 0.2% Triton X-100-PBS for 5 min prior to fluorochrome-conjugated secondary antibody incubation for 1 h at room temperature. Where indicated, F-actin was labelled using TRITC-conjugated phalloidin (Sigma-Aldrich, St. Louis, MO, USA) diluted 1:1500 in PBS for 15 min at room temperature. Finally, coverslips were mounted with VectaShield/DAPI (Vector Laboratories Inc., Burlingame, CA, USA) and further analyzed by confocal laser scanning microscopy (CLSM; Eclipse Ti Series, Nikon Corporation Healthcare Business Unit, Japan) using a 63X (NA = 1.2) oil-immersion objective.

### 4.4. Senescence-Associated β-Galactosidase (SA-β-Gal) Assay

Fibroblasts on coverslips were stained with SA-β-Gal according to the manufacturer’s protocol (Senescent Cell Histochemical Staining Kit, Sigma-Aldrich, St. Louis, MO, USA). SA-β-Gal-positive cells (blue cells) were observed by bright-field microscopy using differential interference contrast.

### 4.5. Western Blotting

Fibroblast cell culture lysates were electrophoresed on 10% SDS–polyacrylamide gels and transferred to an Immobilon-P PVDF membrane (Merck & Co., Rahway, NJ, USA). Membranes were blocked for 1 h in TBST (100 mM Tris-HCL, pH 8.0, 150 mM NaCL, 0.5% (v/v) Tween-20) with low-fat dried milk and then incubated with the corresponding primary antibodies overnight at 4 °C. The protein-specific signal was obtained by using the corresponding secondary antibodies and enhanced luminol-based chemiluminescent substrate for the detection of horseradish peroxidase (HRP) (ECL TM; Amersham Pharmacia, GE Healthcare). Images were acquired by Gel Doc EZ System (Bio-Rad Laboratories Inc., Berkeley, CA, USA), and analyzed for normalization using ImageJ, 1.49 software (Wayne Rasband National Institutes of Health, Kensington, ML, USA. https://imagej.net/).

### 4.6. Immunoprecipitation

Recombinant protein G-agarose beads (10 μL per sample; Invitrogen, Carlsbad, CA, USA) were equilibrated by gentle agitation in lysis buffer (50 mM Tris-HCl, pH 8.0; 150 mM NaCl; 1% Triton X-100; 2 mM Na_3_VO_4_; 25 mM NaF; 10 mM Na_2_MoO_4_; 1 mM PMSF; and 1X complete protease inhibitor mixture) for 2 h at 4 °C. A total of 500 μg of quantified protein lysates per sample was pre-cleared by incubation with the equilibrated beads for 2 h at 4 °C. The beads were then removed by centrifugation, and the resulting supernatant was incubated overnight at 4 °C with 2 μg of anti-β-dystroglycan (β-DG) antibody. As a negative control, parallel incubations were carried out using an irrelevant IgG antibody. Following antibody incubation, fresh equilibrated protein G-agarose beads were added to the lysates and incubated overnight at 4 °C. Immune complexes were collected by centrifugation at 2000× *g* for 3 min and washed three times with 1X PBS. Bound proteins were eluted from the beads by boiling in Laemmli buffer (50 mM Tris-HCl, pH 6.8; 2% SDS; 10% glycerol; 0.1% 2-mercaptoethanol; and 0.001% bromophenol blue) and subsequently analyzed by Western blot.

## 5. Conclusions

In summary, some alterations in the behavior of β-D were identified in NA-Bu-induced senescent fibroblasts and HGPS fibroblasts. These changes include the mislocalization of β-DG to the cytoplasm to a certain extent and the low production of the ICD fragment. In addition, β-DG appears to be sequestered by progerin in HGPS cells, thereby impeding its normal interaction with lamin A.

## Data Availability

The original contributions presented in this study are included in the article/Supplementary Material. Further inquiries can be directed to the corresponding author(s).

## Supplementary Materials

The following supporting information can be downloaded at https://www.mdpi.com/article/10.3390/ijms26167726/s1.

## Abbreviations

The following abbreviations are used in this manuscript:

β-DG	β-distroglycan
NaBu	Sodium butyrate
HGPS	Hutchinson–Gilford progeria syndrome
ICD	Intracellular domain
NE	Nuclear envelope
ETV1	ETS (E twenty-six) variant 1
MMP	Matrix metalloproteinase
UBF	Upstream binding factor
SA-(β-gal)	Senescence-associated β-galactosidase
LB1	Lamin B1
HDAC	Histone deacetylase
NLS	Nuclear import signal
NES	Nuclear export signal
F n/c	Nuclear–cytoplasmic fluorescence intensity ratio
