# Supplementary figures and images for: Analysis of Beta-Dystroglycan in Different Cell Models of Senescence
Source: Int J Mol Sci. 2025 Aug 10;26(16):7726. doi: 10.3390/ijms26167726 (PMC12386449; doi:10.3390/ijms26167726)

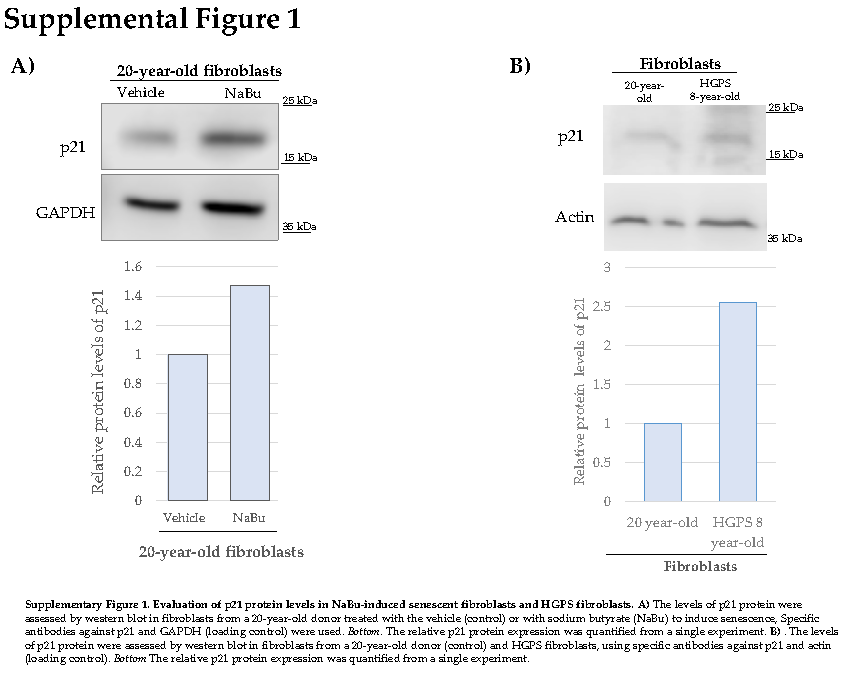

Supplement: Supplementary file 1 [file ijms-26-07726-s001.zip › ijms-3769576-supplementary.png]
